# Focused antenatal care utilization and associated factors in Debre Tabor Town, northwest Ethiopia, 2017

**DOI:** 10.1186/s13104-018-3928-y

**Published:** 2018-11-16

**Authors:** Teshager Workneh Ayalew, Araya Mesfin Nigatu

**Affiliations:** 1 Department of Health Information Technology, Debre Tabor Health Science College, P.O.Box 83, Debre Tabor, Amhara Regional State Ethiopia; 2 Department of Health Informatics, Institute of Public Health, University of Gondar, P.O.Box 196, Gondar, Ethiopia

**Keywords:** Focused antenatal care, Mothers, Debre Tabor, Ethiopia

## Abstract

**Objective:**

Attending antenatal care helps to reduce the occurrence of maternal morbidity and mortality by providing chances for health promotion and information about danger signs, birth preparedness and where to seek care for pregnancy complications. Therefore identifying factors affecting the utilization of focused ANC service is of supreme importance.

**Results:**

A total of 317 mothers who had a history of antenatal care for their last birth during the previous 6 months were included in the study from which 112 (35.3%, 95% CI 30.6, 40.4) of mothers attended focused antenatal care services. Age of mother [AOR = 4.7, 95% CI 1.87, 11.88], Educational status [AOR = 2.5, 95% CI 1.00, 6.19], history of still birth [AOR = 13.1, 95% CI 2.14, 80.20] and planned pregnancy [AOR = 3.7, 95% CI 1.23, 11.12] were found to be major predictors for focused ANC service utilization. Proportion of focused antenatal care was low (35.3%). Age of mother, education, history of stillbirth and planned pregnancy were identified as predictors affecting focused antenatal care service utilization. Encouraging women’s educational status, behavioral change communication at grass root level and improving the capacity and quality of ANC service are some of the recommendations forwarded.

**Electronic supplementary material:**

The online version of this article (10.1186/s13104-018-3928-y) contains supplementary material, which is available to authorized users.

## Introduction

According to World Health Organization WHO 2015 report, globally 303,000 maternal deaths occurred as result of pregnancy and childbirth-related complications each year with 99% (302,000) of them occurred in developing regions, sub-Saharan Africa alone accounts for roughly 66% (201,000), followed by Southern Asia (66,000) [1].

It is known that ANC helps to reduce the occurrence of maternal morbidity and mortality by providing chances for health promotion and information about danger signs, birth preparedness and where to seek care for pregnancy complications [2].

Proper care during pregnancy is important as pregnancy is a crucial time for the health status of the mother and the development of the unborn baby directly by reducing stillbirths and neonatal deaths [3]. Maternal mortality during pregnancy or within 42 days of termination of pregnancy, remains distressingly high in sub-Saharan Africa [4].

WHO recommended that a woman without complications should have at least four antenatal care visits, the first of which should take place during the first trimester [5]. Focused antenatal care (FANC) is personalized care provided to a pregnant woman which emphasizes on the women’s overall health status, her preparation for child birth and readiness for complications or it is timely, friendly, simple safe services to pregnant women [2].

In Africa, the proportion of pregnant women who attended the recommended four or more visits showed remarkable change from time to time [3]; for this education played a major role [6]. As pointed out in EDHS 2014 and EDHS 2016 report same proportion (32%) of mothers received four and above ANC visits [6, 7], but remarkable increment from EDHS 2011 report 19% [8].

Even though, ANC utilization in Ethiopia is very low as compared to developed and most of developing countries, little is known about the proportion and its determinant factors of FANC service utilization. So, the study intends to show current information about the prevalence of FANC and its associated factors in Debre Tabor Town.

## Main text

### Methods

A community based cross-sectional study that assessed the focused antenatal care and associated factors among mothers who gave birth 6 months before the study period in Debre Tabor Town, Amhara Regional State, northwest Ethiopia. The study was conducted between March to June 2017.

The sample size was determined using a single population proportion formula with the assumption; 95% confidence interval, 5% margin of error, 10% estimated non-response rate and taking 25.7% proportion of focused antenatal care service use [9] then, the required sample size was 323.

As there are urban and semi-rural kebeles in the town administration, stratified sampling technique was employed to select study subjects. Women who gave birth for the previous 6 months were participated. Heath extension workers registration book was used as a sampling frame to obtain study participants. All women who gave birth for the previous 6 months prior to the study were registered and then sample was allocated to each Kebele by proportional allocation to their population size. Finally 323 study participants were selected.

Structured and pretested interviewer administered questionnaire including socio-demographic, accessibility, behavioral and obstetrics factors variables ware used. The questionnaire was first prepared in English and then translated into Amharic version for appropriateness in approaching the study participants and then translated back to English by language experts to check its consistency. Three trained diploma nurses and one health officer was assigned as data collectors and supervisor respectively. Training was given for 2 days about the objective, relevance of the study and confidentiality issues.

Data were entered, edited and cleaned using Epi-info version 7 and exported to SPSS version 20 for further statistical analysis. The descriptive analysis such as proportions, percentages, frequency distribution and measure of central tendency were carried out.

Initially, bivariable analysis was performed between dependent and independent variables one by one. Variables found to have an association with the dependent variable less than 0.2 p-value were entered into multivariable binary logistic regression using backward LR method for controlling the possible effects of confounders. Finally the variables which had significant association were identified on the bases of odds ratio with 95% CI. Goodness of fit test was also checked.

Focused ANC utilization is to mean that those pregnant women who attended a minimum of four scheduled ANC visits and receiving all the WHO recommended comprehensive packages by skilled healthcare providers.

Ethical clearance was obtained from the ethical review committee of Fkede Egzee College and permission was also obtained from Debre Tabor Town health office. Study participants were informed about the benefits and risks of the study. Individual participant records were coded on each respective questionnaire and accessed only by the research team members to keep confidentiality.

Training was given for data collectors and supervisor on the objective of the study, data collection procedures, data collecting tools, respondents approach, data confidentiality and respondent’s right prior to the data collection date. The completeness of the questionnaire was checked every other day by the supervisors and principal investigator.

### Results

In this study, a total of 317 women who gave birth 6 months before the study period were participated and responded to the questionnaire, giving a response rate of 98.1%. One hundred twenty-six (39.7%) were in the age group 25–29 years with mean age of 28.2 (SD ± 5.3 years). Majority (94.3%) of respondents were married. In addition, 121 (38.2%) were having educational status of diploma and above and slightly more than half (56.8%) of them were also government employee (Table 1).

**Table 1:** Socio-demographic characteristics of women in Debre Tabor Town northwest Ethiopia, June, 2017 (n = 317)

Variable	Category	Frequency	Percent
Age of mother in years	≤ 24	69	21.8
	25–29	126	39.7
	30–34	62	19.6
	≥ 35	60	18.9
Marital status	Single	3	1.0
	Married	299	94.3
	Divorced	15	4.7
Religion	Orthodox	294	92.7
	Muslim	18	5.7
	Protestant	5	1.6
Educational status of the respondent	Unable to read and write	52	16.4
	Able to read and write	56	17.7
	Primary education (1–8)	18	5.7
	Secondary education (9–12)	70	22.1
	Diploma and above	121	38.2
Occupational status of the respondent	Government employee	108	34.1
	Private worker	10	3.2
	Merchant	37	11.7
	House wife	138	43.5
	Student	20	6.3
	Daily laborer	4	1.3
Ethnicity	Amhara	312	98.5
	Oromo	3	0.9
	Tigrian	2	0.6
Educational status of husband (n = 299)	Unable to read and write	17	5.7
	Able to read and write	55	18.4
	Primary education (1–8)	29	9.7
	Secondary education (9–12)	31	10.4
	Diploma and above	167	55.9
Occupational status of husband (n = 299)	Government employee	162	54.2
	Private worker	18	6.0
	Merchant	102	34.1
	Student	1	0.3
	Daily laborer	16	5.4
Family size	1–3	139	43.8
	4–6	167	52.7
	7–10	11	3.5

The mean age at first pregnancy was 21 (± 3.37 SD) years. About one hundred three (32.5%) of the mothers became pregnant before the age of 20 years and 154 (48.6%) were between the age 20–24. One hundred sixty-two (51.1%) of the mothers had history of two to four pregnancies and 21 (6.6%) of them had history of more than five pregnancies. More than half of the respondents (52.7%) had had 2–4 children and 266 (83.9%) of the mothers had planned pregnancy. In addition, 10 (3.2%) and 12 (3.8%) of the mothers had a history of stillbirth and abortion respectively (Table 2).

**Table 2:** Obstetric characteristics of women in Debre Tabor Town northwest Ethiopia, June, 2017

Variables (n = 317)	Category	FANC N (%)	Not FANC N (%)
Age of mother at first marriage	< 15	3 (13.6)	19 (86.4)
	15–19	31 (38.3)	50 (61.7)
	20–24	48 (32.4)	100 (67.4)
	25–29	30 (45.5)	36 (54.5)
Age of mother at first pregnancy	< 20	34 (33.0)	69 (67.0)
	≥ 20	78 (36.4)	136 (63.6)
Gravidity	1	40 (29.9)	94 (70.1)
	2–4	67 (41.4)	95 (58.6)
	≥ 5	5 (23.8)	16 (76.2)
Parity	1	40 (28.8)	99 (71.2)
	2–4	68 (41.2)	97 (58.8)
	≥ 5	4 (30.8)	9 (69.2)
History of abortion	Yes	8 (66.7)	4 (33.3)
	No	104 (34.1)	201 (98)
History of still birth	Yes	7 (6.3)	3 (1.5)
	No	105 (93.7)	202 (65.9)
Planned pregnancy	Yes	105 (39.5)	161 (60.5)
	No	7 (13.2)	44 (86.85)

Two hundred thirty-five (74.1%) of the respondents obtained information regarding ANC from health institutions and 268 (84.5%) knew attending ANC has the benefit for both the mother and their child. Even though 187 (57.4%) went ANC clinic for regular checkups only 130 (41.0%) of them had knowledge on danger sign during pregnancy (Additional file 1: Table S1). Among mothers who attended ANC for their last pregnancy, 112 (35.3%) visited four and above times. At least one injection of tetanus toxoid 223 (70.3%) was reported by the majority of mothers during their last pregnancy. Two hundred fifty-six (80.8%) of the women obtained laboratory examination. Among mothers who attended ANC, 237 (74.8%) of them waste their time by waiting more than 1 h to get the service. With regard to accessibility, majority 294 (92.7%) of them had better transportation access (Additional file 1: Table S2).

In this study, 112 (35.3%) mothers reported four and above ANC visits as per WHO recommendations (Additional file 2: Figure S1). Regarding their reasons for to attend FANC; health worker’s advice accounts the higher percentage (34.9%) while felt discomfort accounts the least (8.6%) (Additional file 3: Figure S2).

Age of mother, education, history of still birth and planned pregnancy were major predictors for FANC. Multivariable analysis revealed that mothers with age ≥ 35 years (AOR = 4.7, 95% CI 4.87, 11.88) were five times more likely to attend focused antenatal care than with the age group ≤ 24 years. Those respondents who had diploma and above educational status (AOR = 2.5, 95% CI 1.00, 6.19) were three times more likely to attend focused antenatal care when compared to mothers who didn’t read and write. Respondents who had history of still birth (AOR = 13.1, 95% CI 2.14, 80.20) were 13 times more likely to attend focused antenatal care than who didn’t encounter in their life time. Mothers having planned pregnancy (AOR = 3.7, 95% CI 1.23, 11.12) were four times more like to utilize focused antenatal care service compared to those who didn’t plan (Table 3).

**Table 3:** Bivariable and multivariable analysis of focused ANC in Debre Tabor Town, northwest Ethiopia, June, 2017 (n = 317)

Variables	ANC		Crude OR (95% CI)	Adjusted OR (95% CI)
	Non focused (%)	Focused (%)		
Age mother				
≤ 24	42 (60.9)	27 (39.1)	1.00	1.00
25–29	94 (74.6)	32 (25.4)	0.5 (0.28, 0.99)	0.6 (0.28, 1.16)
30–34	43 (69.4)	19 (30.6)	0.7 (0.33, 1.42)	0.6 (0.27, 1.42)
≥ 35	26 (43.3)	34 (56.7)	2.0 (1.01, 4.12)	4.7 (1.87, 11.88)**
Decision of ANC service utilization				
Husband	25 (73.5)	9 (26.5)	1.00	
Wife	74 (83.1)	15 (16.9)	0.56 (0.22, 1.46)	
Both	106 (54.6)	88 (45.4)	2.31 (1.02, 5.19)	
Educational status of mother				
Unable to read and write	37 (71.2)	28 (47.5)	1.00	1.00
Able to read and write	40 (71.4)	57 (55.3)	0.9 (0.43, 2.72)	1.6 (0.16, 1.87)
Primary school (1–8)	15 (83.3)	81 (53.3)	0.5 (0.12, 1.96)	0.3 (0.07, 1.58)
Secondary school (9–12)	49 (70)	3 (30)	1.1 (0.48, 2.33)	1.8 (0.66, 4.98)
Diploma and above	64 (52.9)	57 (47.1)	2.2 (1.09, 4.42)	2.5 (1.00, 6.19)**
Gravidity				
One	94 (70.1)	40 (29.9)	1.00	
Two to four	95 (58.6)	67 (41.4)	1.7 (1.02, 2.69)	
5 and above	16 (76.2)	5 (23.8)	2.6 (0.25, 2.14)	
Parity				
One	99 (71.2)	40 (28.8)	1.00	
Two to four	97 (58.8)	67 (41.4)	1.7 (1.07, 2.81)	
5 and above	9 (69.2)	5 (23.8)	1.1 (0.32, 3.78)	
Source of information for ANC				
Health institution	138 (58.7)	97 (41.3)	11.6 (2.72, 49.48)	
Media/TV, radio	15 (57.7)	14 (42.3)	12.4 (2.38, 61.448)	
Health development army	19 (90.5)	2 (9.5)	1.74 (0.23, 13.35)	
Friend	33 (94.3)	2 (5.7)	1.00	
History of abortion				
Yes	4 (33.3)	8 (66.7)	3.9 (1.14, 13.14)	
No	201 (65.9)	104 (34.1)	1.00	
History of still birth				
Yes	3 (30)	7 (70)	4.5 (1.14, 17.72)	13.1 (2.14, 80.20)**
No	202 (65.8)	105 (34.2)	11.00	1.00
Planned pregnancy				
Yes	161 (60.5)	105 (39.5)	4.1 (1.78, 9.44)	3.7 (1.23, 11.12)**
No	44 (86.3)	7 (13.7)	1.00	1.00

1.00 = reference, Hosmier and Lemesho goodness of fit test = 0.81

### Discussion

According to WHO recommendation, women should receive four and above ANC visits for normal pregnancy [3]. The finding of this study revealed that 35.3% (95% CI 30.6%, 40.4%) of mothers attended FANC service. This finding was consistent with EDHS 2011 report on Amhara Region 34% [8] and Kenya 32% [9], but was higher than previous studies done in Ethiopia districts like East Wolega 14.4% [10], Dejen and Aneded 12% [11] and abroad Kenya 27% [12]. The difference might be due to cultural and awareness differences and currently the government of Ethiopia has given attention by making the service as one of the exempted services.

This finding appeared to be lower than studies done in Ethiopia (Eastern Hararghe zone 38.3% [13] and Holeta town 66% [14]) and abroad Kenya 52% [15], Nigeria 81.5% [16] and Nepal 87% [17]. The probable reason for this might be due to availability of private quality service providing health facilities and also availability of approachable healthcare providers.

In this study mothers whose age ≥ 35 years (AOR = 4.7, 95% CI 4.87, 11.88) were five times more likely to attend FANC service than mother having age group ≤ 24 years. This finding was supported by study done in Wonberma Woreda (Ethiopia) [18] and abroad Nigeria [16]. This might be due to as the age of the mother’s increases, they might have a better knowledge, understanding and experience of pregnancy and pregnancy related complications.

The result of this study also showed that mothers who attended diploma education and above (AOR = 2.5, 95% CI 1.00, 6.19) were three times more likely to attend FANC than those who were unable to read and write. Previous studies done in developing countries have been found that higher educational level was highly associated with much better antenatal care service utilization [8, 16, 19, 20]. The probable reason for this might be due to; women having better educational status are capable of identifying danger signs and easily understand the bad consequence of not attending the recommended antenatal care service.

Highly statistical significant association was also obtained between history of still birth and focused antenatal care service utilization. Mothers who experienced stillbirth before recent pregnancy (AOR = 13.1, 95% CI 2.14, 80.20) were 13 times more likely to utilize antenatal care than those didn’t encountered any yet. This finding was in line with a study done in Ethiopia [19]. The possible explanation for this might be because of those who faced still birth knew the risks associated with it; as a result they might utilize focused antenatal care in order to prevent it and have good pregnancy outcome.

This finding also revealed that planned pregnancy was found to be significantly associated with the utilization of FANC. Pregnant women whose have planned pregnancy (AOR = 3.7, 95% CI 1.23, 11.12) were four times more likely to use focused antenatal care service when compared to those having unplanned pregnancy. This finding is consistent with studies done elsewhere [18, 21]. The possible justification for this might be due to the fact that mother who plan to have a child might want to have a healthy pregnancy and thus might give a great attention for their antenatal care service.

In conclusion, this study proportion was low (35.3%) and 41% had knowledge about danger signs during pregnancy. Age, women’s educational status, history of still birth and planned pregnancy were found to be fundamental predictors of focused antenatal care service utilization. It is well documented that antenatal care visits has the potential in reducing maternal pregnancy related risks and improving newborn’s health if mothers have knowledge on danger sign during pregnancy. Therefore, each pregnant mother should receive the recommended WHO antenatal care visits as it is important to identify risk factors for adverse pregnancy outcomes, both for the mother and their newborn.

### Limitations

Due to the cross-sectional nature of the study, it is difficult to establish cause-effect relationship between dependent and independent variables and social desirability as it was face to face interview.

## Additional files

**Additional file 1: Table S1.** Antenatal care knowledge of mothers in Debre Tabor Town northwest Ethiopia, June, 2017. **Table S2**. ANC service utilization of mothers in Debre Tabor Town northwest Ethiopia, June, 2017.

**Additional file 2: Fig. S1.** Prevalence of FANC among pregnant women attending ANC in Debre Tabor Town, northwest Ethiopia June, 2017.

**Additional file 3: Fig. S2.** Reasons why mothers did attend ANC services in Debre Tabor Town, northwest Ethiopia June, 2017.

## Abbreviations

ANC: ante natal care
EDHS: Ethiopian Demographic and Health Survey
FANC: focused ante natal care
FMOH: Federal Ministry of Health
WHO: World Health Organization
