# Supplementary material for: Focused antenatal care utilization and associated factors in Debre Tabor Town, northwest Ethiopia, 2017
Source: BMC Res Notes. 2018 Nov 16;11:819. doi: 10.1186/s13104-018-3928-y (PMC6240228; doi:10.1186/s13104-018-3928-y)
Supplement: Supplementary file 1 — Additional file 1: Table S1. Antenatal care knowledge of mothers in Debre Tabor Town northwest Ethiopia, June, 2017. Table S2. ANC service utilization of mothers in Debre Tabor Town northwest Ethiopia, June, 2017. [file 13104_2018_3928_MOESM1_ESM.docx]

Table S1: Antenatal care knowledge of mothers in Debre Tabor Town northwest Ethiopia, June, 2017

| **Variables (n=317)** | **Category** | **Frequency** | **Percent** |
| --- | --- | --- | --- |
| Source of information for ANC | Health institution | 235 | 74.1 |
|  | Radio/TV | 26 | 8.2 |
|  | Health development army  Friend | 21  35 | 6.6  11.0 |
| Benefit of ANC | For the pregnant mother | 31 | 9.8 |
|  | For the neonate  for both | 18  268 | 5.7  84.5 |
| Know danger health problem | Yes  No | 130  187 | 41.0  59.0 |
| Good health Worker Approach | Yes  No | 270  47 | 85.2  14.8 |
| When to start ANC visits | 0-3 months  4-6 months  7-9 months | 161  121  35 | 50.8  38.2  11.0 |
| Regular check-ups | Yes | 187 | 57.4 |
|  | No | 135 | 42.6 |

Table S2: ANC service utilization of mothers in Debre Tabor Town northwest Ethiopia, June, 2017

| **Variables (n=317)** | **Category** | **Frequency** | **Percent** |
| --- | --- | --- | --- |
| Number of ANC visits for the last pregnancy | One time  Two times  Three times  Four and above | 44  52  109  112 | 13.9  16.4  34.4  35.3 |
| Preference of heath care provider | Male | 90 | 43.7 |
|  | Female | 109 | 52.9 |
|  | Do not worry | 7 | 3.4 |
| Accessibility of transport service to travel health institution | Yes | 294 | 92.7 |
|  | No | 23 | 7.3 |
| Time to reach health institution | <=60 minute  >=60 minute | 313  4 | 95.6  4.4 |
| Iron during pregnancy | Yes  No | 269  48 | 84.9  15.1 |
| Waiting time to get ANC service | <60 minute  >=60 minute | 237  80 | 74.8  25.2 |
| TT vaccination | Yes  No | 223  94 | 70.3  29.7 |
| Laboratory examination | Yes  No | 256  61 | 80.8  19.2 |
| Blood pressure measured | Yes | 264 | 83.3 |
|  | No | 53 | 29.7 |
