# Supplementary figures and images for: Focused antenatal care utilization and associated factors in Debre Tabor Town, northwest Ethiopia, 2017
Source: BMC Res Notes. 2018 Nov 16;11:819. doi: 10.1186/s13104-018-3928-y (PMC6240228; doi:10.1186/s13104-018-3928-y)

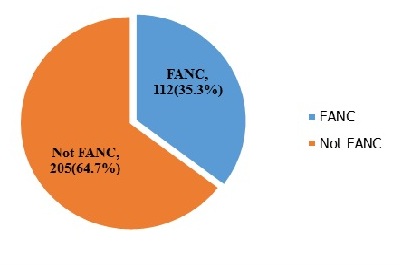

Supplement: Supplementary file 2 — Additional file 2: Fig. S1. Prevalence of FANC among pregnant women attending ANC in Debre Tabor Town, northwest Ethiopia June, 2017. [file 13104_2018_3928_MOESM2_ESM.jpg]

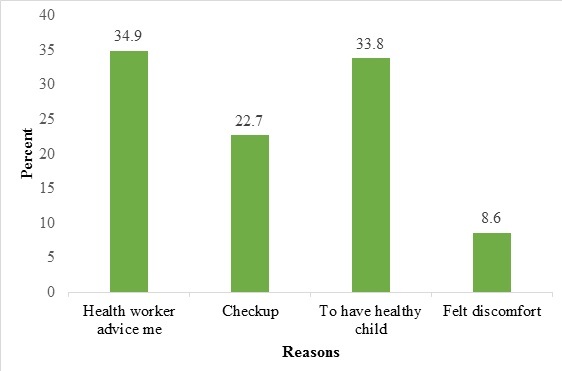

Supplement: Supplementary file 3 — Additional file 3: Fig. S2. Reasons why mothers did attend ANC services in Debre Tabor Town, northwest Ethiopia June, 2017. [file 13104_2018_3928_MOESM3_ESM.jpg]
